# Supplementary material for: The Effects of a Therapeutic Strategy Guided by Lung Ultrasound on 6-Month Outcomes in Patients with Heart Failure: Results from the EPICC Randomized Controlled Trial
Source: J Clin Med. 2022 Aug 22;11(16):4930. doi: 10.3390/jcm11164930 (PMC9409707; doi:10.3390/jcm11164930)
Supplement: Supplementary file 1 [file jcm-11-04930-s001.zip › jcm-1822569-supplementary.pdf]

Suplementary File S1. Futility analysis EPICC trial report.

To evaluate the possibility of continuing with the study, the following futility analysis was proposed, considering the hypotheses:

H0: There are no statistically significant differences between treatments.
H1: There are statistically significant differences between the treatments.

In case of rejecting the null hypothesis, it is concluded that the treatments behave significantly differently, and differences could be reached if the study continues.

If the null hypothesis is not rejected, it is concluded that the treatments behave in a similar way, so it would be futile to continue with it.

Fixed Scenario Elements	
**Distribution**	Asymptotic normal
**Method**	Normal approximation
**Number of Sides**	1
**Alpha**	0.05
**Group 1 Proportion**	0.43
**Group 2 Proportion**	0.2
**Nominal Power**	0.9
**Null Proportion Difference**	0

Computed N per Group	
**Actual Power**	**N per Group**
0.900	68

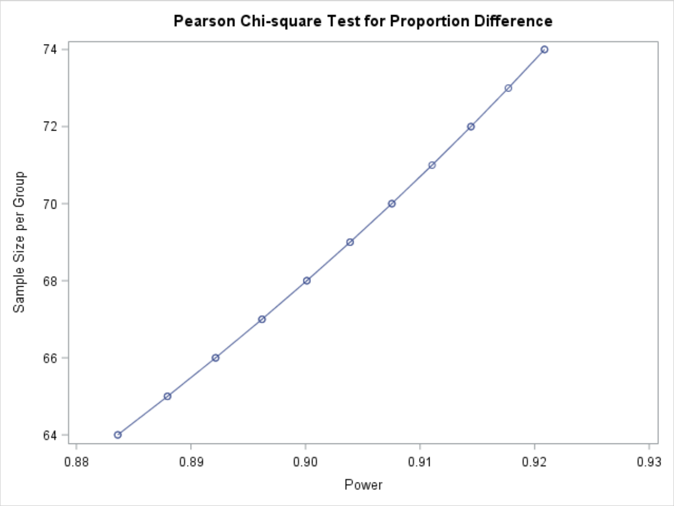

Design Information	
**Statistic Distribution**	Normal
**Boundary Scale**	Standardized Z
**Alternative Hypothesis**	Lower
**Early Stop**	Accept/Reject Null
**Method**	O'Brien-Fleming
**Boundary Key**	Both
**Alternative Reference**	-0.23
**Number of Stages**	2
**Alpha**	0.025
**Beta**	0.1
**Power**	0.9
**Max Information (Percent of Fixed Sample)**	103.3104
**Max Information**	205.2034
**Null Ref ASN (Percent of Fixed Sample)**	68.5966
**Alt Ref ASN (Percent of Fixed Sample)**	84.71245
**Adj Design Alpha**	0.025
**Adj Design Beta**	0.09683
**Adj Design Power**	0.90317
**Adj Design Max Information (Percent of Fixed Sample)**	103.2283

Design Information	
**Adj Design Max Information**	207.3562
**Adj Design Null Ref ASN (Percent of Fixed Sample)**	68.753
**Adj Design Alt Ref ASN (Percent of Fixed Sample)**	84.51339

Method Information								
				Unified Family				
Boundary	Method	Alpha	Beta	Rho	Tau	C	Alternative Reference	Drift
Lower Beta	O'Brien-Fleming	.	0.10000	0.5	0	1.33785	-0.23	-3.29473
Lower Alpha	O'Brien-Fleming	0.02500	.	0.5	0	1.95688	-0.23	-3.29473

Boundary Information (Standardized Z Scale) Null Reference = 0						
				Alternative	Boundary Values	
	Information Level			Reference	Lower	
Stage	Proportion	Actual	N	Lower	Alpha	Beta
1	0.5000	102.6017	83.12791	-2.32973	-2.76744	-0.43772
2	1.0000	205.2034	166.2558	-3.29473	-1.95688	-1.95688

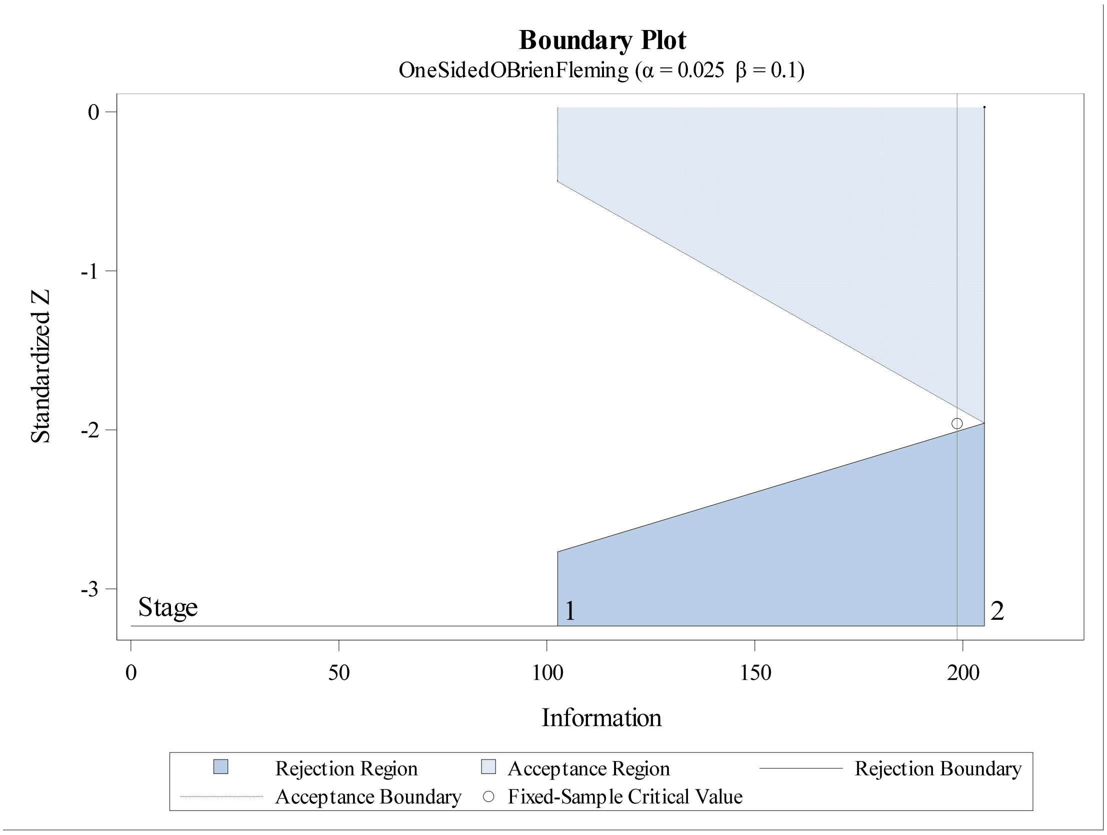

Sample Size Summary	
**Test**	Two-Sample Proportions
**Null Proportion**	0.43
**Proportion (Group A)**	0.2
**Test Statistic**	Z for Proportion
**Reference Proportions**	Alt Ref
**Max Sample Size**	166.2558
**Expected Sample Size (Null Ref)**	110.3914
**Expected Sample Size (Alt Ref)**	136.3264

Sample Sizes (N)<br>Two-Sample Z Test for Proportion Difference								
	Fractional N				Ceiling N			
Stage	N	N(Grp 1)	N(Grp 2)	Information	N	N(Grp 1)	N(Grp 2)	Information
1	83.13	41.56	41.56	102.6	84	42	42	103.7
2	166.26	83.13	83.13	205.2	168	84	84	207.4

Ceiling-Adjusted Design Boundary Information (Standardized Z Scale)<br>Null Reference = 0						
				Alternative	Boundary Values	
	Information Level			Reference	Lower	
Stage	Proportion	Actual	N	Lower	Alpha	Beta
1	0.5000	103.6781	84	-2.34192	-2.76841	-0.42650
2	1.0000	207.3562	168	-3.31197	-1.95756	-1.95756

According to these results, Z test values below -2.76841 result in a rejection of the null hypothesis, while Z values above -0.42650 accept the null hypothesis.

Distribution among groups:

Combi	Group				Total	
	1		2			
	N	%	N	%	N	%
No	26	70.27	31	73.81	57	72.15
Yes	11	29.73	11	26.19	22	27.85
Total	37	100.0	42	100.0	79	100.0

Chi-Square	P-value for Chi-Square	Fisher's Exact Test P-value (2-Tail)
0.122632	0.7262	0.8039

Chi squared value is 0.12, its p-value is 0.7262>0.05, thus null hypothesis of Independence between both variables cannot be rejected

Group 1 vs Group 2

p1Hat	p2Hat	Diff	SE	Z	Side	Pr < Z
0.29730	0.26190	0.035393	0.10107	0.3502	2	0.7262

Conclusions:

Comparing group 1 with Group 2, the statistic Z=0.3501>-0.42650 without detecting statistically significant differences when the contrast statistic was found in the área of acceptance of the null hypothesis of equality between both groups, indicating the stoppage of the study.
